# Wilhelm Uhthoff (1853–1927)

**DOI:** 10.1007/s00415-014-7399-3

**Published:** 2014-06-11

**Authors:** Andrzej Grzybowski, Martyna Pieniążek, Adela Justyńska

**Affiliations:** 1 Department of Ophthalmology, Poznan City Hospital, Poznan, Poland; 2 Chair of Ophthalmology, University of Warmia and Mazury, Olsztyn, Poland; 3 Department of Ophthalmology, Wroclaw Medical University, Wroclaw, Poland; 4 Department of Ophthalmology, Ludwik Rydygier Hospital, Krakow, Poland

**Fig. 1:**
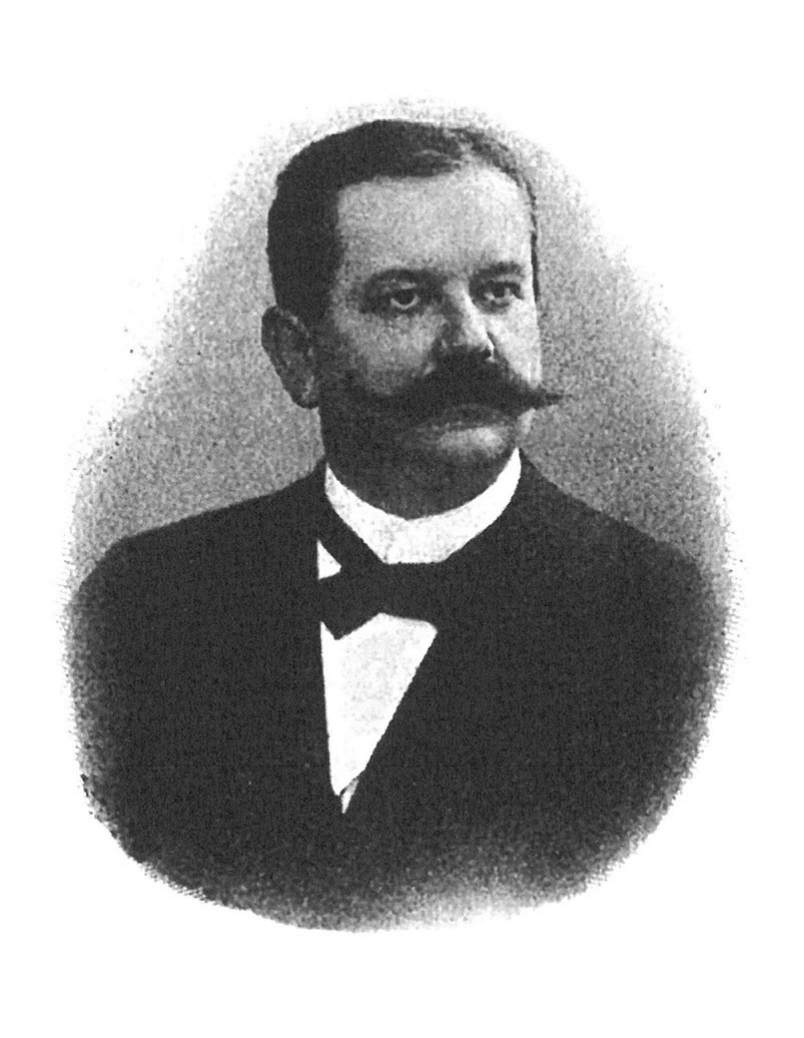
Wilhelm Uhthoff (1853–1927)

Wilhelm Uhthoff (Fig. 1) was a German ophthalmologist, especially interested in neurology. He was born on June 31, 1853, in Klein-Warin in Grand Duchy of Mecklenburg-Schwerin (now Mecklenburg-Vorpommern). He was raised in the countryside, as one of nine siblings. Because of the poor financial situation of his family he could not expect any financial means to support his education. Thus, working 7 days a week was required later on to pay off the loans obtained to cover education-linked expenses [1].

He studied medicine in Tubingen, Gottingen, Rostock and Berlin. He defended his doctoral dissertation in 1877 in Berlin. These years marked the initiation of collaboration with Rudolph Virchow (1821–1902), co-researcher, but also colleague at Breslau University thereafter. In 1878, Uhthoff began ophthalmology residency and became a research assistant at Heinrich Leopold Schoeler’s (1844–1918) clinic in Berlin [2]. Meanwhile, at Helmholtz Physics Institute, he carried out work on light luminance and visual acuity [3]. His interest in neuro-ophthalmology was expressed by establishing consultations at Carl Westphal neurology clinic of Charite Hospital in Berlin [3]. Among his co-workers Adolf Wallenberg (1862–1949), Hermann Oppenheim (1858–1919), Carl Moehli (1849–1919) and Ernst Siemerling (1857–1931) can be listed [2]. Uhthoff shared a bacteriological research interest with his eldest student, Theodor Axenfeld (1867–1930). In 1885, he obtained senior doctor lecturer title and 5 years later was appointed Ordinary Professor to the Chair of Ophthalmology in Marburg.

In 1896, he accepted the position of the Head of the University Eye Clinic and moved to Breslau. Advancing his academic career, he was nominated for rector of Friedrich Wilhelm Silesian University (Schlesische Friedrich-Wilhelms-Universität zu Breslau) and, subsequently, University of Breslau in 1908.

Uhthoff was a devoted researcher and lecturer—many case reports of his authorship established the basis of modern neuro-ophthalmology. His research covered multiple fields, including eye anatomy and pathology, relation between light luminance and visual acuity, alcohol-induced loss of vision, color perception, visual acuity, sclera anatomy, cornea and conjunctiva pathology as well as difference in ophthalmologists’ and neurologists’ perception [4].

In 1890, he observed and described a novel symptom of reversible blurriness of vision after a strenuous physical exercise among multiple sclerosis patients. This symptom is known today as Uhthoff’s phenomenon (known also as Uhthoff sign or syndrome).

In 1915, he described ophthalmological signs and symptoms of brain tumors, improving the picture of Foster-Kennedy syndrome [5]. During the 1st World War, while examining soldiers with head injuries, in cooperation with Sir Gordon Morgan Holmes (1876–1965), he worked on cortical representation of retinal areas [6]. He also described retinal detachment management, ophthalmological manifestations of intoxications and was an advocate for blind patients [4].

Uhthoff retired in 1923, however, he did not cease his scientific activity, but continued working on publications on acquired research material. He died suddenly of a heart attack as a complication of influenza on March 21, 1927, in Breslau [3].
